# Enhanced ultrasound imaging and anti-tumor *in vivo* properties of Span–polyethylene glycol with folic acid–carbon nanotube–paclitaxel multifunctional microbubbles†

**DOI:** 10.1039/c9ra06437k

**Published:** 2019-10-31

**Authors:** Jie Zhang, Limei Song, Shujing Zhou, Ming Hu, Yufeng Jiao, Yang Teng, Ying Wang, Xiangyu Zhang

**Affiliations:** Pharmacy College, Jiamusi University Jiamusi 154007 China; College of Materials Science & Engineering, Jiamusi University Jiamusi 154007 China

## Abstract

With Span and polyethylene glycol (PEG) as the membrane material, the as-prepared folate–carbon nanotube–paclitaxel (FA–CNT–PTX) complex was added to the reaction system under sound vibration cavitation and Span–PEG with FA–CNT–PTX microbubbles was obtained. The maximum tolerating dose of the obtained composite microbubbles on Kunming mice was determined by acute toxicity test. Utilizing the breast cancer tumor model in the nude mice to assess the anti-tumor activity *in vivo*, the inhibition effect of the composite microbubbles on tumor growth was analyzed by recording the weight and tumor volume of the nude mice. HE staining observations, the immunohistochemistry method, and TUNEL were, respectively, used to examine the inhibition effect of the composite microbubbles on breast cancer tumors in the nude mice. The ultrasound imaging effects and the changes in the peak intensities of the composite microbubbles were inspected using a Doppler color ultrasound imaging system. The experimental results showed that the maximum tolerated dose of the composite microbubbles was 3500 mg kg^−1^, indicating that the composite microbubbles had low toxicity and good biocompatibility. The composite microbubbles could reach the breast cancer tumor *via* a targeting factor, and then hindered the tumor growth by inhibiting the proliferation of tumor cells and inducing apoptosis of the tumor cells. The composite microbubbles contributed toward enhancing the ultrasound signal and improved the resolution of the ultrasound images and extended the imaging time. Also, the addition of CNTs in the composite microbubbles could enhance the ultrasound contrast. Simultaneously, the peak intensity at the tumor was significantly reduced after the treatment.

## Abstract 2

With Span and polyethylene glycol (PEG) as the membrane material, the as-prepared folate–carbon nanotube–paclitaxel (FA–CNT–PTX) complex was added to the reaction system under sound vibration cavitation and Span–PEG with FA–CNT–PTX microbubbles was obtained.

## Graphical abstract

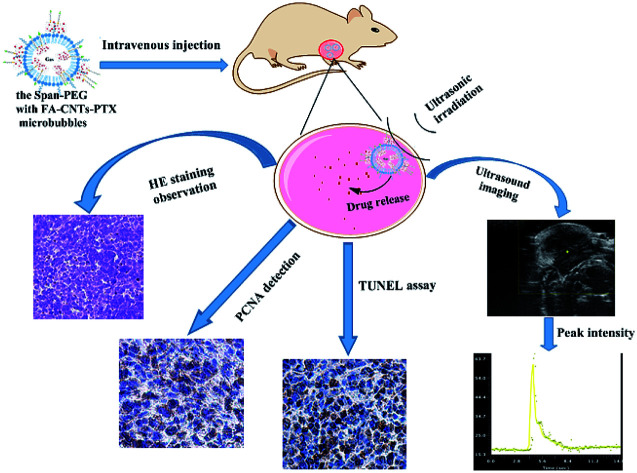

## 1. Introduction

Nowadays, malignant tumors represent an increasingly common serious threat to human health and are second only to cardiovascular disease for causing death. Among such tumors, breast cancer is a widespread cancer worldwide, threatening women's bodies and lives.^1,2^ Some research data have showed that breast cancer tends to occur at a younger age, and has gradually become a global health risk and economic burden in the world, so there is clearly an urgent need to find new treatment methods.^3–6^ Chemotherapy can effectively prolong the survival of patients with malignant tumors, while adverse reactions related to chemotherapy treatment have been the main problem limiting its clinical application.^7–10^ The development of drug carriers with low toxicity and long-term release could effectively help alleviate many of the problems with chemotherapy.

Ultrasound-targeted drug-loaded microbubble destruction (UTMD) is a new targeted drug-delivery method. With acoustic microbubble loading of the drug, local ultrasonic irradiation could realize the dual effects of sustained release and targeted drug delivery.^11^ Simultaneously, ultrasonic irradiation can promote the endocytosis of tissue cells and produce a sound pore effect, which would increase the drug uptake of target tissues without destroying cells.^12^ UTMD can provide a safe and effective way to reduce systemic adverse reactions in the treatment of tumors and other diseases.^13,14^

Currently, paclitaxel (PTX) is a commonly used chemotherapy drug in the clinical treatment of ovarian cancer and breast cancer, and it can be combined with tubulin to form stable microbundles, thereby interfering with mitosis and inhibiting the proliferation of cancer cells.^15^ PTX is a lipophilic and hydrophobic compound. As a result, polyoxyethylene castor oil is used to help PTX dissolve in PTX injections, both in markets at home and abroad, but it has problems that this can cause allergic reactions. L. Milas *et al.*^16^ prepared ultrasonic microbubbles containing PTX with phospholipid as an encapsulating material, and reported its toxicity was 10 times less than the current PTX injection, and also that it had ultrasonic imaging ability and could be broken by ultrasound. Studies showed that ultrasound-mediated PTX microbubbles could effectively inhibit the growth of ovarian cancer, breast cancer, and pancreatic cancer, and that the tumor volume could be significantly reduced after such administration.^17–19^ However, in most studies, microbubbles have been reported as having some shortcomings, such as limited dosage, poor stability, and sudden drug release, which directly affect the safety of the contrast agents used.^20^

Carbon nanotubes (CNTs), with a unique hollow structure and high specific surface area, are one of the most advanced nanocarriers for the efficient delivery of drugs and biological molecules.^21^ They can combine with different drugs, biomolecules, and nanoparticles by non-covalent or covalent binding. After their functional modification, carbon nanotubes can have even better biocompatibility and lower toxicity, and their high surface loading-rate and solubilization, targeting, and other functions can be improved.^22–24^ At the same time, carbon nanotubes are also useful as a new and powerful diagnostic imaging tool. A few studies have applied carbon nanotubes to ultrasonic imaging and obtained enhanced ultrasound imaging and good biological adaptability.^25–28^

Based on the above reasons, CNTs were used as a drug carrier and folic acid (FA) as a targeting factor in this study, where paclitaxel (PTX) was first loaded onto CNTs with FA, and then the as-prepared FA–CNTs–PTX compound was incorporated into a Span–PEG membrane material. The prepared Span–PEG with FA–CNTs–PTX microbubbles is a multifunctional ultrasound contrast agent that enables enhanced ultrasound imaging and targeting therapy.

## 2. Experimental methods

### 2.1 Preparation of Span–PEG with FA–CNT–PTX microbubbles

Carboxyl carbon nanotubes (CNTs–COOH) were first prepared by mixed-acid heating reflux. Folic acid (FA) and EDC were dissolved in dimethyl sulfoxide (DMSO) at a mass ratio of 1 : 4; then they were introduced into a chitosan–acetic acid solution under the dark at room temperature. The acetone solution was added into this system at the reaction end until the resultant solution was completely precipitated. The precipitation was respectively washed by DMSO and double distilled water and the product obtained after freeze-drying was a folic acid and chitosan (FA–CS) compound.

CNTs–COOH and FA–CS acetic acid aqueous solution were mixed and reacted in the dark at room temperature. The resultant precipitation was washed till neutral and the FA–CNTs targeting intermediate was obtained. PTX ethanol solution was dropwise added into the ethanol dispersed system of FA–CNTs in the dark. The targeting drug-loaded complex (FA–CNTs–PTX) was obtained after the washing and freeze-drying. Next, 450 mg Span, 450 mg PEG (*M*_W_ = 1500), and 900 mg NaCl were fully ground and mixed; then dispersed in phosphate buffer solution (PBS). Next, 30 mg FA–CNTs–PTX complex was introduced and the acoustic cavitation process was performed by using the ultrasonic cell crushing apparatus, with N_2_ simultaneously injected into the system. The obtained suspension was centrifuged, and the supernatant liquid was collected and washed with the same amount of PBS in the separatory funnel. Then the microbubbles in the middle layer were selected and freeze-dried, obtaining Span–PEG with FA–CNTs–PTX microbubbles.

### 2.2 Acute toxicity study

All animal procedures were performed in accordance with the Guidelines for Care and Use of Laboratory Animals of Jiamusi University and approved by the Animal Ethics Committee of Jiamusi, Heilongjiang Province. Here, 9 Kunming mice were randomly divided into 3 groups, and Span–PEG with a FA–CNT–PTX microbubble suspension at the concentrations of 1000, 3000, and 5000 mg kg^−1^ were injected into the abdominal cavities of each group of mice, respectively. Then observation was sustained for 72 h, and the number of dead animals was recorded. The experiment was repeated according to the dose-ratio until the doses at the 3/3 group and 0/3 group were found, and the minimum dose of 100% death (*D*_m_) and the maximum dose of 0% death (*D*_n_) were obtained. Based on the experimental objective necessity and the principle of saving animals, the *D*_n_ value was used as the maximum dose of the safety limit experiment for the mice. Next, 20 Kunming mice were randomly selected and intraperitoneally injected with the maximum dose. The toxicity and weight change of the mice were observed for 7 days after injection. The multiple of the maximum tolerance dose was determined by the following formula.
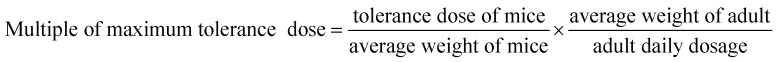


### 2.3 Establishment and grouping of the nude mice with the breast cancer model

RPMI 1640 containing 20% fetal bovine serum and 1% penicillin–streptomycin were used to cultivate the breast cancer cells MCF-7 under 37 °C, 5% CO_2_ (v%), and fully saturated humidity. When the cell growth was in a good condition, the cells were digested by 0.25% trypsin and resuspended by the complete medium. The cell concentration was adjusted to 2.5 ×10^7^ number mL^−1^ by PBS. Next, 100 μL cell suspension was hypodermically injected into the armpit position of the mice. It was noteworthy that the tumors in the mice reached an average size of 80 mm^3^ after the underarm injection. Next, 12 of the tumor nude mice were randomly divided into four groups and used in the following experiments: (a) Span–PEG microbubble control group, (b) Span–PEG with FA–CNTs microbubble group, (c) Span–PEG with CNTs–PTX microbubble group, (d) Span–PEG with FA–CNTs–PTX microbubble group.

### 2.4 Inhibition study on the breast cancer tumors of the nude mouse

The tail veins of the tumor-bearing mice were injected with different composite microbubbles in Section 2.3, and the dose was 350 mg kg^−1^. Each group of tumor-bearing mice was administrated once every 4 days, for a treatment cycle of 16 days.

During the treatment, the weight changes of every group of mice were recorded after the administration, and the length diameter (*a*) and short diameter (*b*) of the transplanted tumors were measured with a Vernier caliper to calculate the tumor volume (*V*) and tumor volume inhibition rate (IR).
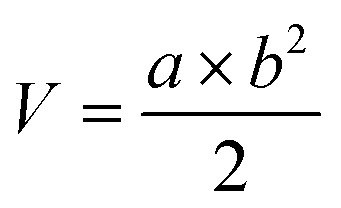

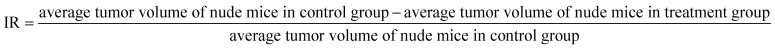


After the treatment, the nude mice were killed, and the tumors were immediately took out and fixed in 10% neutral buffered formalin overnight, and a 4 mm thickness paraffin-embedded section was used for HE staining observation.

The expression of PCNA in breast cancer tumor cells was detected using the peroxidase SABC kit. Five vision fields per section were randomly photographed under biopsy light microscopy at ×500 magnification. The number of PCNA positive cells in 500 tumor cells per field was counted, and the proliferation index (PI) was calculated according to the following formula.
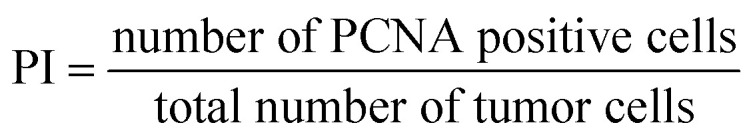


TUNEL was used to detect apoptosis of the breast cancer tumor cells. Here, 5 high-fold visual fields were randomly observed in the biopsy, and the number of TUNEL-positive cells per 500 tumor cells was counted, and the apoptosis index (AI) was calculated by the following formula.
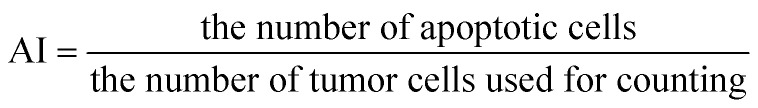


### 2.5 Ultrasound imagings *in vivo* and *in vitro*

In a tank, a sponge layer was used as a sound-absorbing layer. The tank was filled with the degassed double distilled water, keeping it at a constant temperature of 37 °C, and the water surface was covered by a plastic film (no bubbles). A medical silicone tube was employed to simulate the blood vessel and was immersed in the water tank, different microbubbles were respectively injected into the medical silicone tubes at a concentration of 0.5 mg mL^−1^. An ultrasonic probe of a Doppler color ultrasound imaging system was placed under the water surface to conduct the ultrasonic imaging for the silicone tubes.

In the *in vivo* anti-tumor experiment under Section 2.4, after each group of nude mice were respectively injected with the different composite microbubbles at the first and last times, and the Doppler color ultrasound imaging system was employed to examine the tumors of the nude mice. The ultrasound parameters remained the same during the whole experiment process, and the effects of the ultrasound imaging and variations of the peak intensities were timely recorded.

## 3. Results and discussions

**Fig. 1:**
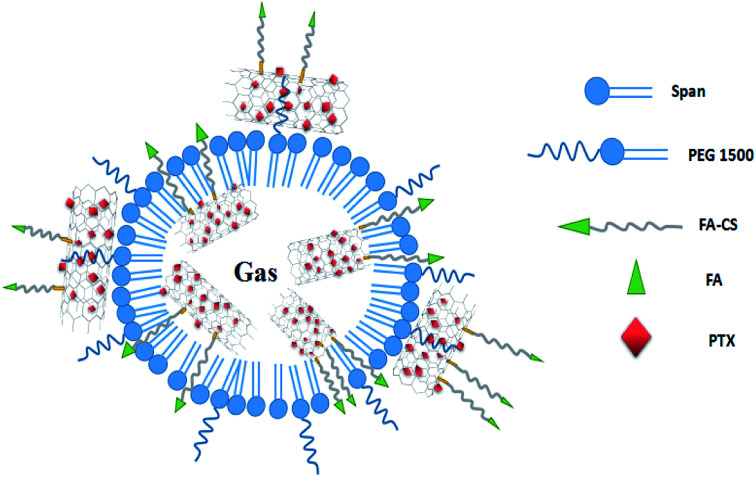
Simulation diagram of the ultrasound contrast agent structure of the composite microbubbles.^29,30^

**Fig. 2:**
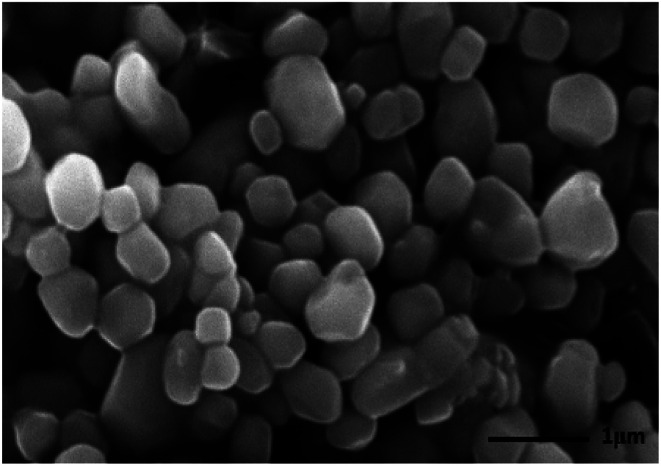
Micromorphology of the composite microbubbles (×10 000).

**Fig. 3:**
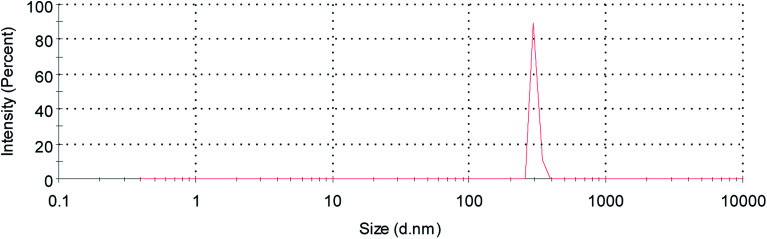
Size distribution of the composite microbubbles.

**Fig. 4:**
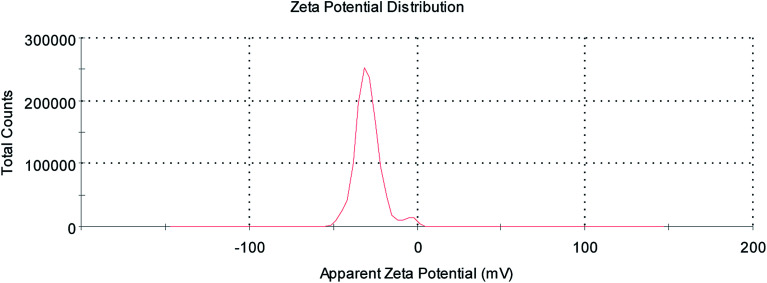
Apparent zeta potential of the composite microbubbles.

As described in the previous work, the structure of the composite microbubbles is shown in Fig. 1, where it can be seen that the prepared Span–PEG with FA–CNTs–PTX microbubbles was a hollow sphere, and the FA–CNTs–PTX delivery system was embedded in the Span–PEG membrane.^29,30^Fig. 2 and 3 show, respectively, the SEM and size distribution of the composite microbubbles. It can be seen from Fig. 2 and 3 that the composite microbubbles had a smooth surface and uniform size, and the average particle size was 442 nm, which means they could pass through the tumor microvascular network, which is more conducive to the combination of the microbubbles and the tumor. After the ultrasonic irradiation, the ultrasonic contrast agent microbubbles produced a cavitation effect, whereby the microbubbles break up and release the carried drugs.^31,32^ As shown in Fig. 4, the average zeta potential of the composite microbubbles was −27 mV, showing the structure was stable.

### 3.1 Acute toxicity effect

An administration dosage of the Span–PEG with FA–CNTs–PTX microbubbles was selected in the range of 1000–5000 mg kg^−1^. The acute toxicity symptoms did not occur at the dose of 3000 mg kg^−1^; however, when the dose reached 5000 mg kg^−1^, the Kunming mice began to appear to show the death phenomenon and eventually all died. Therefore, *D*_n_ and *D*_m_ were further chosen between 3000 and 5000 mg kg^−1^, as the acute toxicity did not appear at the dose of 3500 mg kg^−1^, but it began to happen and even mice died over 3500 mg kg^−1^, and indeed all the mice died when the dose reached 4500 mg kg^−1^. As a result, *D*_n_ was determined to 3500 mg kg^−1^ and *D*_m_ was 4500 mg kg^−1^. A safety limit experiment was conducted with the *D*_n_ value as the maximum tolerated dose. Kunming mice were selected and intraperitoneally injected in the experiment. From Table 1, it can be seen that the weights of the selected mice normally increased, while death and other toxic symptoms did not emerge. It can be seen that the Span–PEG with FA–CNTs–PTX microbubbles were safe at the maximum dose, and LD_50_ was greater than the maximum dose (3500 mg kg^−1^). Specifically, a daily dose of such a clinical ultrasound contrast agent for an adult was about 10 mg kg^−1^, and the multiple of the maximum tolerated dose of the mice was 350 times, more 100 times than that of humans.

**Table 1:** Acute toxicity test results of Span–PEG with FA–CNTs–PTX microbubbles

Animal number	Weight (kg)	Dose (mg kg^−1^)	Sex	Death within 7 days (±)	Acute toxicity (±)
1	0.025	3500	Male	—	—
2	0.028	3500	Male	—	—
3	0.021	3500	Male	—	—
4	0.025	3500	Male	—	—
5	0.022	3500	Male	—	—
6	0.023	3500	Male	—	—
7	0.024	3500	Male	—	—
8	0.023	3500	Male	—	—
9	0.022	3500	Male	—	—
10	0.026	3500	Male	—	—
11	0.022	3500	Female	—	—
12	0.025	3500	Female	—	—
13	0.021	3500	Female	—	—
14	0.019	3500	Female	—	—
15	0.022	3500	Female	—	—
16	0.022	3500	Female	—	—
17	0.024	3500	Female	—	—
18	0.020	3500	Female	—	—
19	0.021	3500	Female	—	—
20	0.021	3500	Female	—	—

### 3.2 Inhibition effect on the breast cancer tumor of the nude mice

**Fig. 5:**
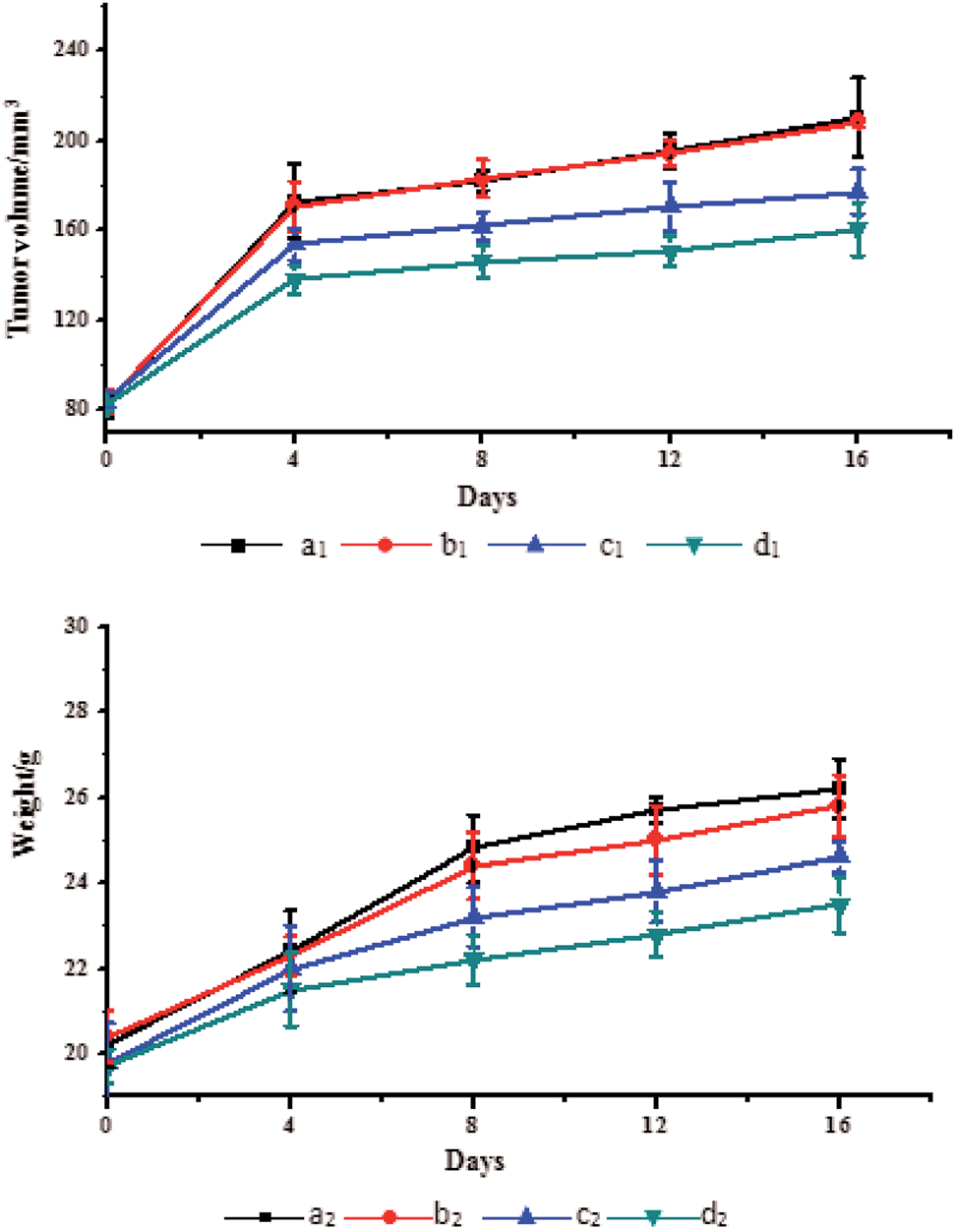
Tumor volumes and weights of the nude mice in each group during treatment: (a) Span–PEG microbubble control group, (b) Span–PEG with FA–CNTs microbubble group, (c) Span–PEG with CNT–PTX microbubble group, (d) Span–PEG with FA–CNT–PTX microbubble group (subscript 1 denotes the tumor volume, subscript 2 denotes the weight of the nude mice).

The tumor volume changes of the nude mice in each group during the treatment are shown in Fig. 5. It can be seen from Fig. 5 that Span–PEG with CNTs–PTX microbubbles (Fig. 5(c_1_)) and Span–PEG with FA–CNTs–PTX microbubbles (Fig. 5(d_1_)) had different inhibitory effects on the tumor growth in comparison with the Span–PEG microbubble control group (Fig. 5(a_1_)), while Span–PEG with FA–CNTs microbubbles (Fig. 5(b_1_)) had no therapeutic effect on the tumors. Specifically, after a treatment of 16 days, the tumor volume for the Span–PEG microbubble control group (Fig. 5(a_1_)) was 210.5 mm^3^, and that of the Span–PEG with FA–CNTs microbubble group (Fig. 5(b_1_)) was 208.4 mm^3^, where the no difference between the two groups was because the Span–PEG microbubbles and Span–PEG with FA–CNTs microbubbles were not loaded with the anti-tumor drug. On the contrary, as PTX was loaded on the CNTs, the tumor volumes of the nude mice in the Span–PEG with CNT–PTX microbubble group (Fig. 5(c_1_)) and Span–PEG with FA–CNTs–PTX microbubble group (Fig. 5(d_1_)) were, respectively, 177.5 mm^3^ and 160.6 mm^3^, and the inhibition rates on the tumor volume were 23.7% and 15.7%. Furthermore, as FA was connected to the Span–PEG with FA–CNT–PTX microbubbles, the composite microbubbles were more conducive to bind with the tumors. Therefore, Span–PEG with FA–CNT–PTX microbubbles had a stronger inhibitory effect on the tumor than Span–PEG with CNT–PTX microbubbles.

The weight changes of the nude mice in each group during the treatment are shown in Fig. 5. It can be seen from Fig. 5 that the nude mice weights in each group showed an increasing trend with the extension of the treatment time, and the specific sequence was the Span–PEG microbubble control group (Fig. 5(a_2_)) > Span–PEG with FA–CNT microbubble group (Fig. 5(b_2_)) > Span–PEG with CNT–PTX microbubble group (Fig. 5(c_2_)) > Span–PEG with FA–CNT–PTX microbubble group (Fig. 5(d_2_)). Among these, the weight gain of the nude mouse for the Span–PEG with FA–CNT–PTX microbubble group was the least, attributed to its targeted therapeutic effect on the tumors.

**Fig. 6:**
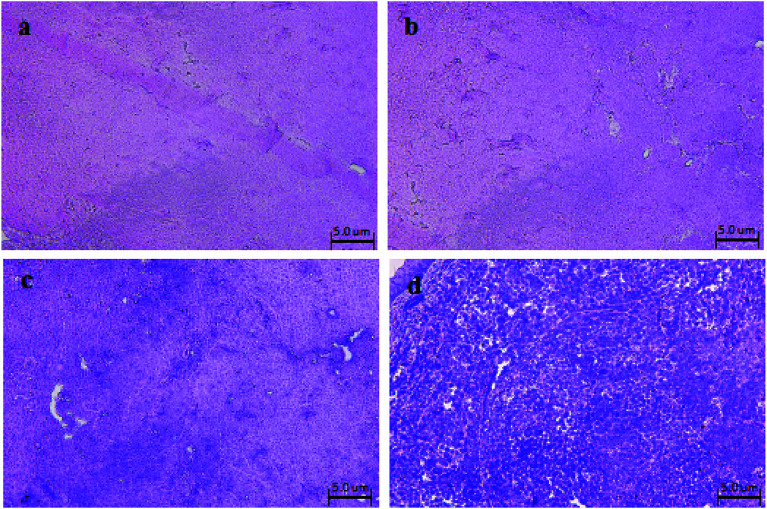
HE staining sections of nude mice tumors in each group after treatment: (a) Span–PEG microbubble control group, (b) Span–PEG with FA–CNT microbubble group, (c) Span–PEG with CNT–PTX microbubble group, (d) Span–PEG with FA–CNT–PTX microbubble group.

After 16 days of treatment, the tumor tissues of the nude mice for the Span–PEG microbubbles control group, Span–PEG with FA–CNT microbubble group, Span–PEG with CNT–PTX microbubble group, and Span–PEG with FA–CNT–PTX microbubble group were removed. The tumors were round, oval, or lobulated, frequently invading the skin, and they had abundant blood vessels and no lymph nodes or metastasis to other organs. HE staining sections of the tumor tissues in each group were observed by light microscopy, as shown in Fig. 6. From Fig. 6, for the Span–PEG microbubble control group (Fig. 6(a)) and Span–PEG with FA–CNT microbubble group (Fig. 6(b)), the tumor cells showed a dense, disordered state and chord-like structure, large cells, abundant cytoplasm and a pathological nuclear fission presented, and heteromorphism phenomenon were obvious. For the Span–PEG with FA–CNTs–PTX microbubble group (Fig. 6(d)) and Span–PEG with CNT–PTX microbubble group (Fig. 6(c)), the tumor cells became sparse and their number decreased, and some cells showed pyknosis and turned into isolated individuals, which indicated that the two composite microbubbles had a therapeutic effect on the tumor. In addition, the number of tumor cells for the Span–PEG with FA–CNT–PTX microbubble group (Fig. 6(d)) was the least and the growth of tumor cells was inhibited, resulting from the preferable anti-tumor activity of Span–PEG with FA–CNT–PTX microbubbles.

**Fig. 7:**
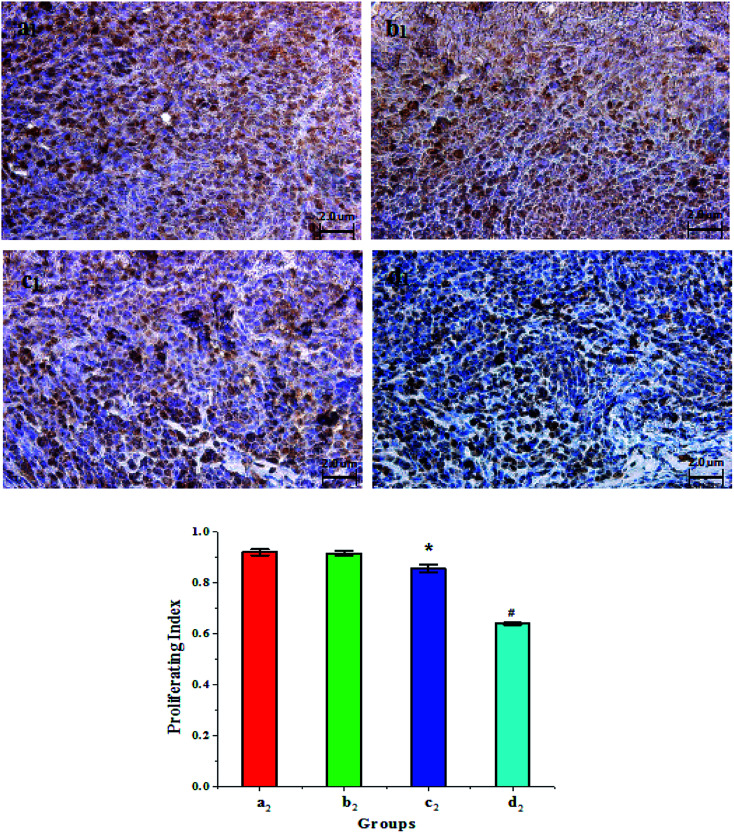
PCNA detection and proliferation index (PI) of the tumor cells in each group after treatment: (a) Span–PEG microbubble control group, (b) Span–PEG with FA–CNT microbubble group, (c) Span–PEG with CNT–PTX microbubble group, (d) Span–PEG with FA–CNT–PTX microbubble group (subscript 1 denotes PCNA detection; subscript 2 denotes proliferation index, *p < 0.05, #*p* < 0.01).

PCNA expression of the tumor cells in each group of nude mice after treatment was detected by the immunohistochemistry method, as shown in Fig. 7. PCNA expression is related to the clinical stage, differentiation, and prognosis of tumor patients. Therefore, PCNA can reflect the synthesis state of DNA in the tumor cells and an indicator of whether the tumor cells have proliferative activity. PCNA-positive cells were defined according to the cell nuclei being stained to a uniform brownish yellow, while the cytoplasm and cell membrane were not stained. As shown in Fig. 7, the number of PCNA-positive cells for the Span–PEG with FA–CNT microbubble group (Fig. 7(b_1_)) was approximately equal with that of the Span–PEG microbubble control group (Fig. 7(a)) however, the Span–PEG with CNT–PTX microbubble group (Fig. 7(c_1_)) was less and the Span–PEG with FA–CNT–PTX microbubble group (Fig. 7(d_1_)) was the least.

Proliferation indexes (PI) of the tumor cells in each group after treatment are shown in Fig. 7. The proliferation index of Span–PEG with FA–CNT–PTX microbubble (Fig. 7(d_2_)) was 0.640 ± 0.004 and that of the Span–PEG microbubble control group (Fig. 7(a_2_)) was 0.921 ± 0.012, and a significant difference between the two groups was obvious (*p* < 0.01). The proliferation index of the Span–PEG with FA–CNT microbubble group (Fig. 7(b_2_)) was 0.916 ± 0.011, which was not statistically significant different in comparison with the Span–PEG microbubble control group (*p* > 0.05). However, the proliferation index of the Span–PEG with CNT–PTX microbubble group (Fig. 7(c_2_)) was 0.856 ± 0.015 and showed a significant difference in comparison with the Span–PEG microbubble control group (*p* < 0.05).

The above results indicated that the proliferation activity of cancer cells for the Span–PEG microbubbles control group was high, while the proliferation activities of cancer cells for the Span–PEG with CNT–PTX microbubble group and Span–PEG with FA–CNT–PTX microbubble group decreased, with the reduction in the Span–PEG with FA–CNT–PTX microbubble group being especially obvious. It can be concluded that Span–PEG with FA–CNTs–PTX microbubbles could enable the targeted inhibition of the proliferation of the tumor cells.

**Fig. 8:**
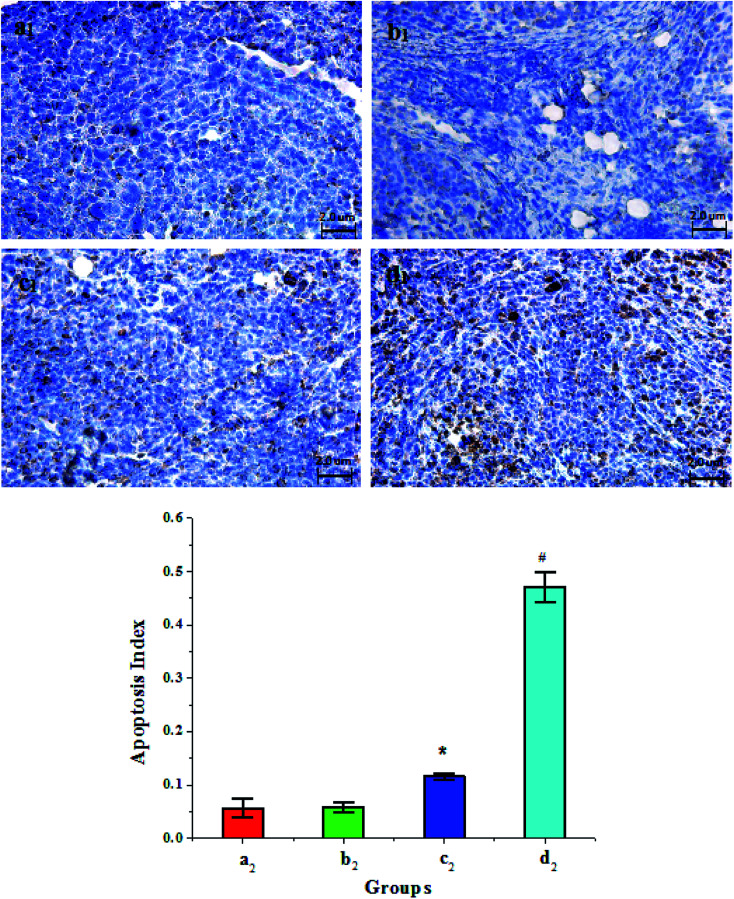
Apoptosis condition and apoptosis index (AI) of tumor cells in each group after treatment: (a) Span–PEG microbubble control group, (b) Span–PEG with FA–CNT microbubble group, (c) Span–PEG with CNT–PTX microbubble group, (d) Span–PEG with FA–CNT–PTX microbubble group (subscript 1 denotes apoptosis condition; subscript 2 denotes apoptosis index, *p < 0.05, #*p* < 0.01).

The apoptosis level of tumor cells after treatment was detected by the TUNEL method. The nuclei of normal tumor cells were blue and those of apoptotic tumor cells were brown. As shown in Fig. 8, there were almost no apoptotic cells in the Span–PEG microbubble control group (Fig. 8(a_1_)) and Span–PEG with FA–CNT microbubble group (Fig. 8(b_1_)), while a little amount of apoptotic cells were found in the Span–PEG with CNTs–PTX microbubble group (Fig. 8(c_1_)), and the Span–PEG with FA–CNT–PTX microbubble group presented abundant brown apoptotic cells (Fig. 8(d_1_)), suggesting that the inhibitory effect of the composite microbubbles on the tumor cells was from PTX.

The apoptosis indexes (AI) of tumor cells in each group are shown in Fig. 8. From Fig. 8, the AI of the Span–PEG with FA–CNT microbubble group (Fig. 8(b_2_)) was 0.058 ± 0.0089 and that of the Span–PEG microbubble control group was 0.056 ± 0.018 (Fig. 8(a_2_)), and there was no significant difference between the two groups (*p* > 0.05). The AI of the Span–PEG with CNT–PTX microbubble group was 0.116 ± 0.006, which was significantly higher than the Span–PEG microbubble control group (*p* < 0.05). However, the AI of the Span–PEG with FA–CNTs–PTX microbubble group (Fig. 8(d_2_)) was 0.471 ± 0.0289, which revealed a significant difference in comparison with the Span–PEG microbubble control group (*p* < 0.01). Moreover, taking FA as the target factor, Span–PEG with FA–CNT–PTX microbubbles could accelerate the apoptosis process in comparison with Span–PEG with CNT–PTX microbubbles, which further proved that the drug-carrying microbubbles modified by FA could achieve the targeted delivery of the drug, and the increase in drug concentration at the tumor site improved the effect of Span–PEG with FA–CNTs–PTX microbubbles in accelerating the apoptosis.

### 3.3 Ultrasound imaging effects *in vivo* and *in vitro*

**Fig. 9:**
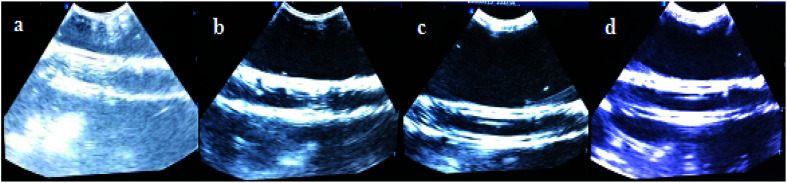
Ultrasound images of the different groups *in vitro* for: (a) normal saline, (b) Span–PEG microbubbles, (c) Span–PEG with CNT microbubbles, (d) Span–PEG with FA–CNT–PTX microbubbles.

Fig. 9 shows the ultrasonic images of the silicone tubes after the injection of saline, Span–PEG microbubbles, Span–PEG with CNT microbubbles, and Span–PEG with FA–CNTs–PTX microbubbles. Compared with the normal saline group (Fig. 9(a)), the ultrasonic signals of the Span–PEG microbubble group (Fig. 9(b)), Span–PEG with CNT microbubble group (Fig. 9(c)), and Span–PEG with FA–CNT–PTX microbubble group (Fig. 9(d)) were significantly enhanced, and the ultrasonic signals were uniform in the tubes. In addition, dense and fine dots with a strong echo were presented and there was no obvious sound shadow behind the image for these three composite microbubbles. In Fig. 9(b)–(d), the ultrasound imaging signals for the Span–PEG microbubbles, Span–PEG with CNT microbubbles, and Span–PEG with FA–CNTs–PTX microbubbles increased in this order *in vitro*. It's worth noting that the enhanced ultrasound imaging effect of Span–PEG with CNTs microbubbles was more obvious than that of Span–PEG microbubbles, while the ultrasonic signal of Span–PEG with CNTs microbubbles was similar to that of Span–PEG with FA–CNTs–PTX microbubbles. It can be concluded that the addition of CNTs in the composite microbubbles effectively enhanced the ultrasound imaging. At the same time, the ultrasonic signals in the tube were not quenched during the ultrasound imaging, which proved that the composite microbubbles had good stability.

**Fig. 10:**
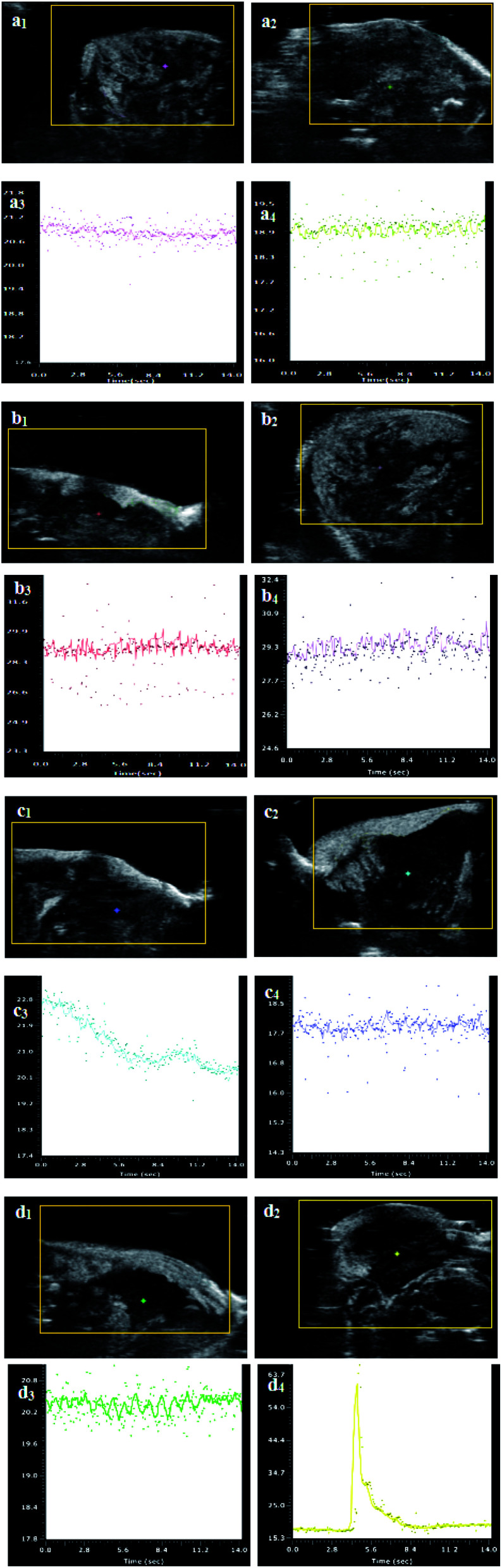
Ultrasound images and contrast peak intensities of nude mice tumors in each group before and after treatment: (a) Span–PEG microbubble control group, (b) Span–PEG with FA–CNTs microbubble group, (c) Span–PEG with CNTs–PTX microbubble group, (d) Span–PEG with FA–CNT–PTX microbubble group (subscript 1 and 2, respectively, denote the ultrasound images before and after treatment, subscript 3 and 4, respectively, denote the contrast peak intensities before and after treatment).

Before and after treatment, the ultrasound imaging results of the mice tumors in each group are shown in Fig. 10. After the Span–PEG microbubbles, Span–PEG with FA–CNT microbubbles, Span–PEG with CNT–PTX microbubbles, and Span–PEG with FA–CNT–PTX microbubbles were, respectively, injected into the tail veins of the nude mice, the blood vessels in the tumor site of the nude mice were rapidly filled and the ultrasound signals enhanced, and it was found that the definition of the ultrasound image in the tumor area was remarkably improved. For the Span–PEG with FA–CNT microbubbles (Fig. 10(b_1_) and (b_2_)), Span–PEG with CNT–PTX microbubbles (Fig. 10(c_1_) and (c_2_)), and Span–PEG with FA–CNT–PTX microbubbles (Fig. 10(d_1_) and (d_2_)), their ultrasound signal intensities were similar, and the definitions of their ultrasound imaging were much higher than for the Span–PEG microbubbles (Fig. 10(a_1_) and (a_2_)). As a result, the ultrasound imaging effect of the composite microbubbles could be significantly enhanced by adding CNTs into the microbubbles, which was in agreement with the results found in the ultrasonic imaging *in vitro*. In addition, the Span–PEG with FA–CNTs microbubbles (Fig. 10(b_1_) and (b_2_)) and Span–PEG with FA–CNTs–PTX microbubbles (Fig. 10(d_1_) and (d_2_)) showed a long ultrasound time at the tumor site in comparison with the Span–PEG microbubbles (Fig. 10(a_1_) and (a_2_)) and Span–PEG with CNTs–PTX microbubbles (Fig. 10(c_1_) and (c_2_)). This was because the targeting factor FA was connected to the FA receptor that was excessively generated at the tumor, so that the composite microbubbles accumulated at the tumor and extended the ultrasonic imaging time. Fig. 10(a_1_), (b_1_), (c_1_), (d_1_) and 10(a_2_), (b_2_), (c_2_), (d_2_), respectively, show the ultrasound imaging effects of the nude mice tumors in each group before and after treatment. The tumor volumes of the nude mice after treatment for the Span–PEG microbubble control group and Span–PEG with FA–CNT microbubble group (Fig. 10(a_2_) and (b_2_)) were significantly increased in comparison with those before treatment (Fig. 10(a_1_) and (b_1_)) because the no-drug composite microbubbles did not have a therapeutic effect on the tumor. However, for the Span–PEG with CNT–PTX microbubble group and Span–PEG with FA–CNT–PTX microbubble group, the tumor volumes of the nude mice after treatment (Fig. 10(d_1_) and (d_2_)) increased a little in comparison with those before treatment (Fig. 10(c_1_) and (c_2_)), indicating that the Span–PEG with CNT–PTX microbubbles and Span–PEG with FA–CNT–PTX microbubbles had an inhibitory effect on the tumor growth, which was consistent with the results in Fig. 8.

Fig. 10 shows the contrast peak intensities of the nude mice tumors before and after treatment. It can be seen from the ultrasound images in each group before treatment (Fig. 10(a_3_)–(d_3_)) that the tumors had abundant bloodstreams and showed a high perfusion state, as the peak intensities were high. It could be found that there were no significant changes in the peak intensities of the ultrasound images after treatment for the Span–PEG microbubble control group (Fig. 10(a_4_)) and Span–PEG with FA–CNTs microbubble group (Fig. 10(b_4_)). For the Span–PEG with CNT–PTX microbubbles (Fig. 10(c_4_)) and Span–PEG with FA–CNT–PTX microbubbles (Fig. 10(d_4_)) after treatment, patchy filling defects could be seen in the tumor areas, and there was no obvious perfusion state and the peak intensity was reduced at some tumor areas. It was noteworthy that the decrease in peak intensity was the most obvious for the Span–PEG with FA–CNT–PTX microbubbles. This was because the composite microbubbles as the cavitation nuclei increased the concentration of cavitation nuclei at the tumor sites through FA targeting, so the cavitation effect was enhanced and the required ultrasound energy was reduced at this phase. At the same time, the ultrasound process could stimulate the microbubbles to produce the cavitation effect, which directly affected the microvascular networks that supplied the nutrition for the tumor tissues. As a result, the microvessels in the tumor and its surrounding tissues were damaged, which blocked the neovascularization in the tumor region. More drug-loaded microbubbles could enter into the tumors and a targeted delivery of drug was thus achieved, so the tumor growth was inhibited.

## 4. Conclusions

For the prepared Span–PEG with FA–CNT–PTX ultrasound contrast agent microbubbles, the maximum tolerance ratio of mice was 350 times. This further indicated that the application of CNTs in the ultrasound diagnostic reagent was safe. Span–PEG with FA–CNT–PTX microbubbles could reach the tumors by targeting, then inhibited the proliferation of tumor cells, induced the apoptosis of tumor cells, and ultimately inhibited the tumor growth. As a kind of diagnostic ultrasound agent, Span–PEG with FA–CNT–PTX microbubbles provided a stronger ultrasound signal and clearer ultrasound image *in vivo* and *in vitro*.

## Conflicts of interest

There are no conflicts to declare.

## Supplementary Material

RA-009-C9RA06437K-s001
