# Correction: Enhanced ultrasound imaging and anti-tumor *in vivo* properties of Span–polyethylene glycol with folic acid–carbon nanotube–paclitaxel multifunctional microbubbles

**DOI:** 10.1039/d6ra90040b

**Published:** 2026-04-09

**Authors:** Jie Zhang, Limei Song, Shujing Zhou, Ming Hu, Yufeng Jiao, Yang Teng, Ying Wang, Xiangyu Zhang

**Affiliations:** a Pharmacy College, Jiamusi University Jiamusi 154007 China; b College of Materials Science & Engineering, Jiamusi University Jiamusi 154007 China

## Abstract

Correction for “Enhanced ultrasound imaging and anti-tumor *in vivo* properties of Span–polyethylene glycol with folic acid–carbon nanotube–paclitaxel multifunctional microbubbles” by Jie Zhang *et al.*, *RSC Adv.*, 2019, **9**, 35345–35355, https://www.doi.org/10.1039/C9RA06437K.

The authors would like to bring attention to duplicated images in Fig. 9a in *RSC Adv.*, 2019, **9**, 35345–35355 (https://www.doi.org/10.1039/C9RA06437K) and Fig. 5a in *ACS Omega*, 2019, **4**(3), 4691–4696 (https://www.doi.org/10.1021/acsomega.8b03403).

Both figures represent blank control groups in saline solution. The authors regret that a reference to the *ACS Omega* paper was not included in the *RSC Advances* paper. This Correction serves to add a reference to the caption of Fig. 9a of *RSC Adv.*, 2019, **9**, 35345–35355, as displayed herein.

**Fig. 1:**
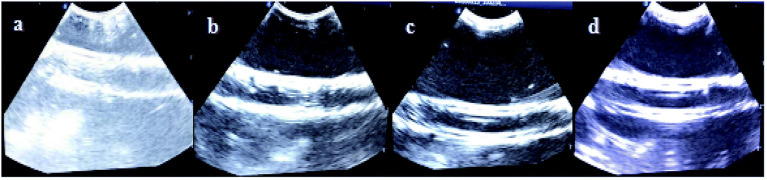
Ultrasound images of the different groups *in vitro* for: (a) normal saline,^1^ (b) span–PEG microbubbles, (c) span–PEG with CNT microbubbles, (d) span–PEG with FA–CNT–PTX microbubbles.

In the *ACS Omega* paper, the authors prepared polylactic acid (PLA) composite microbubbles (FA/DOX/GO/DOX/PLA) using graphene oxide (GO) as the carrier to load folic acid (FA) and doxorubicin (DOX).

In the *RSC Advances* paper, the authors used Span and polyethylene glycol (PEG) as membrane materials, and Span–PEG composited FA–CNT–PTX microbubbles were obtained through the addition of folate–carbon nanotube–paclitaxel (FA–CNT–PTX) into the reaction system.

The experimental conditions were the same and the same instrument was used to investigate the *in vitro* ultrasonic imaging effects of the two composite microbubbles at the same time. Fig. 9a in *RSC Adv.*, 2019, **9**, 35345–35355 (https://www.doi.org/10.1039/C9RA06437K) and Fig. 5a in *ACS Omega*, 2019, **4**(3), 4691–4696 (https://www.doi.org/10.1021/acsomega.8b03403) both represent saline groups for the *in vitro* ultrasound imaging simulation experiment. Therefore, one image was selected as the blank control.

An independent expert has considered the authors’ explanation and has concluded that it is consistent with the discussions and conclusions presented, though they noted that the authors should have recorded separate control images for each experiment.

The Royal Society of Chemistry apologises for these errors and any consequent inconvenience to authors and readers.
