# Supplementary material for: Enhanced ultrasound imaging and anti-tumor in vivo properties of Span–polyethylene glycol with folic acid–carbon nanotube–paclitaxel multifunctional microbubbles
Source: RSC Adv. 2019 Oct 31;9(61):35345–55. doi: 10.1039/c9ra06437k (PMC9074749; doi:10.1039/c9ra06437k)
Supplement: RA-009-C9RA06437K-s001 [file RA-009-C9RA06437K-s001.pdf]

Electronic Supplementary Material (ESI) for RSC Advances.
This journal is © The Royal Society of Chemistry 2019

Reagents and materials

Paclitaxel, Folic acid (Chengdu Must biological technology Co. LTD, China); Chitosan (CS, Qingdao Hailan biological products Co. LTD, China), Carbon nanotube (Guangzhou Yenrui Chemical Co. LTD, China); Carbodiimide hydrochloride (EDC)(analytical pure, Sigma Inc, America); Sorbitan monostearate (Span) and Polyethylene glycol (PEG)(Tianjin Comiao Chemical Reagent Co. LTD, China).

Nude mice, Breast cancer cells MCF-7 (Wuxi Innovate Biopharmaceutical Technology Co. LTD, China); Kunming mice (Experimental Animals Center of Jiamusi University, China); RPMI 1640 medium (Shanghai Bioleaf Biotech Co. LTD, China); TUNEL kit, peroxidase SABC kit, PCNA expressed antibody (Shanghai Abcam Trading Co. LTD, China).

Instruments

Ultrasonic cell crushing apparatus (FS1500T, Shanghai Shenxi ultrasonic instrument Co. LTD. China); Doppler color ultrasound imaging system (S40, Wuhan Liangkang Medical device Co., LTD, China); Fluorescence microscope (JSXF-JSC009, Shanghai optical instrument factory, China); Microscope (DM14000, Germany Leica LTD, Germany); Refrigerated centrifuge (KUBOTA 5500, Beijing Dongxuntiandi Medical instrument Co., LTD, China)
